# Accounting for squared coherence marginally improves the discriminative power of frequency domain cerebral autoregulation markers in surgical aortic valve replacement patients

**DOI:** 10.1007/s11517-026-03558-4

**Published:** 2026-04-02

**Authors:** Francesca Gelpi, Beatrice Cairo, Vlasta Bari, Beatrice De Maria, Pavandeep Singh, Martina Anguissola, Carlo De Vincentiis, Marianna Volpe, Raffaella Molfetta, Marco Ranucci, Alberto Porta

**Affiliations:** 1 Department of Cardiothoracic, Vascular Anesthesia and Intensive Care, IRCCS Policlinico San Donato, San Donato Milanese, Milan, Italy; 2 Department of Biomedical Sciences for Health, University of Milan, Milan, Italy; 3 IRCCS Istituti Clinici Scientifici Maugeri, Milan, Italy; 4 Department of Cardiac Surgery, IRCCS Policlinico San Donato, Milan, Italy; 5 Department of Cardiac Rehabilitation, IRCCS Policlinico San Donato, Milan, Italy; 6 Università degli Studi di Milano, Dipartimento di Scienze Biomediche per la Salute IRCCS Policlinico San Donato, Laboratorio di Modellistica di Sistemi Complessi, Via R. Morandi 30, 20097 San Donato Milanese, Milano Italy

**Keywords:** Cerebrovascular control, blood pressure variability, mean cerebral blood flow, transfer function, active standing, surrogate analysis

## Abstract

The dynamic component of the cerebral autoregulation (CA) is characterized via the transfer function (TF) from mean arterial pressure (MAP) to mean cerebral blood velocity (MCBv), acquired noninvasively via transcranial Doppler device. TF analysis theoretically requires the significance of squared coherence (K^2^). However, the practical relevance of assessing the significance of K^2^ is unclear. The study compares K^2^, TF phase (TFP), and TF gain (TFG) assessed via a moving window approach over all the frames regardless of K^2^ and after exclusion of those frames with K^2^ below the significance threshold. The approach is based on the generation of uncoupled surrogate pairs that preserve distribution and power spectral density of the original series while being fully uncoupled. MAP and MCBv were recorded in 24 patients (age: 61 ± 14 yrs, 18 males, 6 females) scheduled for surgical aortic valve replacement (SAVR). Data was acquired at supine resting and during active standing the day before the procedure (PRE) and within 7 days after (POST). In SAVR population we found that: (i) the practical relevance of accounting for the significance of K^2^ to discriminate time points and experimental conditions is marginal especially at time scales typical of CA; (ii) CA is preserved in both PRE and POST and this conclusion holds irrespective of K^2^ and its significance. Cerebrovascular control is so complex that the concomitant action of multiple regulatory mechanisms might have reduced the efficacy of the application of the criterion based on K^2^ significance in SAVR population.

**Graphical Abstract:**

**Figure:**
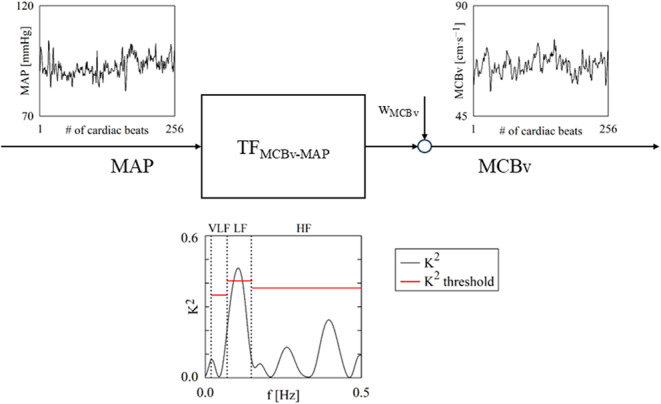
Accounting for squared coherence (K^2^) marginally improves the discriminative power of transfer function (TF) markers of cerebral autoregulation derived from spontaneous fluctuations of mean arterial pressure (MAP) and mean cerebral blood velocity (MCBv) in surgical aortic valve replacement patients

**Supplementary Information:**

The online version contains supplementary material available at 10.1007/s11517-026-03558-4.

## Introduction

Cerebral autoregulation (CA) refers to a regulatory mechanism that aims to maintain approximately constant average cerebral blood flow (CBF) over a relatively large range of average arterial pressure (AP) values under steady-state condition [1]. The effect of CA was highlighted by combining populations featuring different steady-state levels of AP [1]. The practical value of CA was later proven individually by characterizing its dynamic component via the application of a sudden change of AP [2] or the analysis of the spontaneous beat-by-beat fluctuations of mean AP (MAP) [3] and mean CBF (MCBF). It is believed that, if the dynamic component of the CA was functioning, a variation of MAP would not produce important variations of MCBF after a rapidly vanishing transient because of the derivative characteristic of its frequency response [4]. Under this hypothesis transfer function (TF) analysis was proposed as a tool to describe the dynamic component of CA. Continuous noninvasive monitoring of AP and cerebral blood velocity (CBv) via volume-clamp technology [5] and transcranial Doppler (TCD) ultrasonography [6] respectively, allows the computation of MAP and mean CBv (MCBv) on a beat-to-beat basis with a time resolution adequate to the TF estimation. Therefore, TF approach applied to MAP and MCBv beat-to-beat variability series has become a standard approach for the characterization of CA [7–22].

Theoretically TF-based analysis requires the fulfilment of several hypotheses [4]. In addition to assuming stationarity, linearity of input and output series, and linearity and time-invariance of their relationship, one extra prerequisite is the significant association between them [23–31]. The reliability of the TF estimate degrades while decreasing the level of association and TF estimate becomes completely unreliable when the two series a fully uncoupled. The degree of association between input and output is usually quantified through squared coherence (K^2^) and the situation of full uncoupling is decided by setting of a threshold above which K^2^ is deemed to be significant [24–31]. Several procedures have been devised to set this threshold: (i) the selection of an arbitrary and high value of K^2^, usually 0.5 [24]; (ii) the computation of a theoretical value of K^2^ according to the degrees of freedom of the analysis [3, 20, 25]; (iii) the computation of a heuristic K^2^ function via the generation of a set of artificial series built by preserving the dynamic features of the two original series while destroying any relationship between them [26–31]. The procedure based on generation of synthetic surrogates is more frequently utilized in association with the parametric approach to the estimate of K^2^ function [26–28, 31], even though examples of application of the method in association with non-parametric estimates can be found [29, 30]. Different levels of K^2^, including K^2^ values indicating no MCBv-MAP association, might contribute to poor reproducibility of TF gain (TFG) and TF phase (TFP) [12, 15, 16]. In addition, it has been suggested [3, 20] that the use of a criterion based on a threshold on K^2^ might limit the arbitrariness of the selection of the frame of analysis within the experimental session, thus again contributing to the improvement of the reproducibility of the analysis.

The impact of accounting for the level of association between spontaneous variations of physiological variables when estimating TF-based indexes has been evaluated in the context of the characterization of baroreflex control [32]. It was observed that testing the prerequisite of significant K^2^, although necessary from a methodological standpoint, did not produce any practical advantage [32], likely because of the presence of other additional confounding factors, e.g. respiration [33, 34], and the complexity of the baroreflex that it is not only limited to its cardiac arm [35, 36]. In the context of the analysis of CA, the present study was designed to check the practical relevance of removing from the set of analyzed MAP and MCBv sequences the ones whose level of K^2^ was below the threshold of significance.

Thus, the aim of the present study is to characterize CA via TF-based indexes computed after removal of MAP and MCBv sequences featuring an insufficient degree of MCBv-MAP dynamic interactions according to a surrogate data approach [31]. These results are compared with those obtained without applying the prerequisite of a significance level association between MAP and MCBv variability series, namely by considering all the sequences regardless of K^2^. To avoid the arbitrariness of the selection of the sequence within the experimental session we propose a moving window analysis iterating the surrogate approach. The approach was applied to data recording in a SAVR cohort. This population is particularly insightful to judge the relevance of accounting for the prerequisite of significant K^2^. Indeed, autonomic dysfunction, baroreflex depression and chronic cerebral hypoperfusion might have induced an impairment of CA in SAVR population but its detection might require the application of a reproducible criterion for improving the statistical power of the analysis. In previous studies [17, 37] that did not apply the criterion to test the significance of the K^2^ no effect of the orthostatic challenge in the SAVR group on TF indexes was found, especially at slow time scales that are the most frequently involved in CA.

## Methods

### Compliance with ethical standards

The study adhered to the principles of the Declaration of Helsinki for medical research involving humans. The study was approved by the ethical review board of the San Raffaele Hospital, Milan, Italy (approval number: 68/int/2018; approval date: 5 April 2018) and authorized by the IRCCS Policlinico San Donato, San Donato Milanese, Italy (authorization date: 13 April 2018). All subjects gave written informed consent before performing the experimental sessions.

### Population and experimental protocol

The sample was a subset of a larger database built at the IRCCS Policlinico San Donato to prospectively assess the autonomic response, baroreflex control and cerebrovascular regulation in patients undergoing SAVR [17, 37] and to compare results with those obtained from a cohort of patients undergoing a less invasive procedure such as the transcatheter aortic valve implantation [22]. Briefly, we considered 24 patients (age: 61 ± 14 yrs, 18 males) undergoing SAVR at the IRCCS Policlinico San Donato, San Donato Milanese, Italy. The patients included in this study were chosen within the entire cohort of SAVR patients because they featured the longest recordings. In previous studies in SAVR groups of similar sizes [17, 22] either STAND or surgical procedure did not produce any CA modifications. Therefore, in the present study we utilized a group of comparable size to test that the conclusion about the preservation of CA might change whether segments with values of K^2^ below the threshold of significance are discarded. The size of the SAVR group allowed the preoperative detection of the expected decrease of the mean value of MCBv in response to an orthostatic challenge [38–40]. Demographic and clinical data of enrolled patients are reported in Table 1. Exclusion criteria were history of atrial fibrillation, overt autonomic nervous system pathologies or cerebrovascular diseases. Patients were instructed to avoid caffeine and alcoholic beverages in the 24 h preceding the test. All experimental procedures took place in the morning in a temperature-controlled room. Subjects were instrumented to continuously monitor electrocardiogram (ECG) from lead II (BioAmp FE132, ADInstruments, Australia), non-invasive finger AP by volume-clamp photoplethysmography (CNAP Monitor 500, CNSystems, Austria), respiration (RESP) via a thoracic piezoelectric belt (ADInstruments, Australia) and CBv via a TCD device (Multi-Dop X, DWL, San Juan Capistrano, CA, USA) from the left or right middle cerebral artery (MCA). Signals were sampled at 400 Hz through a commercial acquisition system (Power Lab, ADInstruments, Australia). Signals were recorded before surgery (PRE), namely one day before SAVR procedure, and after surgery (POST), namely within 7 days after SAVR procedure. All the subjects were instrumented and instructed to lie down on the same ambulatory bed for 10 min before starting recording. After this period of acclimatation with setup and position, ECG, AP and CBv were recorded for 10 min at rest in supine position (REST). Following the REST phase, participants were then asked to first sit down and then stand up within a few seconds. Signals were acquired for additional 10 min during active standing (STAND). None of the patients had ambulatory difficulties and exhibited presyncope signs during STAND. AP signal was cross-calibrated using systolic AP (SAP) and diastolic AP (DAP) obtained through a sphygmomanometer before starting the REST session. CBv was measured by the TCD device according to the frequency shift between emitted and reflected ultrasound waves. At REST, the signals were recorded in all subjects in both PRE and POST. During STAND, the signals were recorded in 22 out of 24 subjects in PRE and in 19 out of 24 subjects in POST, due to difficulties either for the patient to maintain the orthostatic position or for the operator to insonate the MCA.

**Table 1:** Clinical and demographic markers of enrolled SAVR patients

Index	SAVR (*n* = 24)
Age [yrs]	61 ± 14
Gender [male]	18 (75)
Weight [kg]	76 ± 12.5
BMI [kg·m^− 2^]	26.1 ± 3.4
Congestive heart failure	1 (4)
Recent myocardial infarction	0 (0)
Previous cerebrovascular events	1 (4)
LVEF [%]	58.6 ± 10.1
Diabetes	3 (13)
COPD	0 (0)
Serum creatinine [mg·dl^− 1^]	0.99 ± 0.35
Hypertension	9 (38)
HCT [%]	42.2 ± 4.6
ACE inhibitors	8 (33)
Beta-blockers	14 (58)
Diuretics	4 (16)
Calcium antagonists	1 (4)
Antiarrhythmic drugs	1 (4)
Combined intervention	10 (41)
EuroSCORE II	1.9 ± 1.8
CPB time [minutes]	89.5 ± 30.7
Nadir temperature on CPB [°C]	33.8 ± 1.5
Catecholamine administration	0 (0)
Mechanical ventilation time [hours]	11.1 ± 5.7
ICU stay [days]	1.4 ± 0.7
Hospital stay [days]	7 ± 1.5
Postoperative atrial fibrillation	8 (33)
Postoperative arrhythmias	1 (4)
Postoperative stroke	0 (0)
Postoperative acute kidney injury	0 (0)

*SAVR* surgical aortic valve replacement; *BMI* body mass index; *LVEF* left ventricular ejection fraction; *COPD* chronic obstructive pulmonary disease; *HCT* hematocrit; *ACE* angiotensin converting enzyme; *EuroSCORE* European System for Cardiac Operative Risk Evaluation; *CPB* cardiopulmonary bypass; *ICU* intensive care unit. Continuous data are presented as mean ± standard deviation and categorical data as number (percentage)

### Extraction of beat-to-beat variability series

We followed a standard procedure [41] for the computation of variability series of heart period (HP), SAP, DAP, MAP, and MCBv. Variables were extracted on a beat-to-beat basis and never interpolated in the time domain [17, 37]. The procedure is reported in Section “*Computation of beat-to-beat variability series*” in the file Supplementary Material. Analyses during STAND were carried out three minutes after the onset of the session to avoid transitory adjustments of variables. The lengths of the series were not significantly different across either experimental conditions or time points, being in PRE 446 ± 92 beats at REST and 404 ± 60 beats during STAND, and in POST 479 ± 118 beats at REST and 441 ± 83 beats during STAND.

### Time- and frequency-domain univariate variability markers

In the time domain we computed the mean µ and variance σ^2^ of the beat-to-beat series of HP, SAP, DAP, MAP and MCBv. They were labelled µ_HP_, σ^2^_HP_, µ_SAP_, σ^2^_SAP_, µ_DAP_, σ^2^_DAP_, µ_MAP_, σ^2^_MAP_, µ_MCBv_ and σ^2^_MCBv_, respectively and expressed in ms, ms^2^, mmHg, mmHg^2^, mmHg, mmHg^2^, mmHg, mmHg^2^, cm·s^− 1^, and cm^2^·s^− 2^, respectively.

Frequency-domain univariate markers were computed over MAP and MCBv variability series according to an autoregressive modeling approach [42]. Details of the method are reported in the Section “*Frequency-domain univariate CA markers*” in the file Supplementary Material. Following the indications reported in [3, 28], spectral indexes were computed in very low frequency (VLF) band (from 0.02 to 0.07 Hz), low frequency (LF) band (from 0.07 to 0.15 Hz) and high frequency (HF) band (from 0.15 to 0.5 Hz band). The VLF, LF and HF powers of the MAP and MCBv series were expressed in absolute units, namely mmHg^2^ and cm^2^·s^− 2^ respectively, and labelled VLF_MAP_, VLF_MCBv_, LF_MAP_, LF_MCBv_, HF_MAP_ and HF_MCBv_.

### Frequency-domain bivariate variability markers

TF-based markers are commonly utilized to characterize CA [4]. TF was computed from MAP to MCBv according to a bivariate autoregressive approach [33]. Details of the method are reported in the Section “*Frequency-domain bivariate CA markers*” in the file Supplementary Material. K^2^, TFP, and TFG were sampled at the peak of K^2^ detected in the VLF, LF, and HF bands. K^2^, TFP, and TFG were expressed in dimensionless units, radians (rad), and cm·s^− 1^·mmHg^− 1^ respectively. The indexes were labelled K^2^_VLF_, TFP_VLF_, TFG_VLF_, K^2^_LF_, TFP_LF_, TFG_LF_, K^2^_HF_, TFP_HF_, and TFG_HF_.

### Assessing the K^2^ significance via a running threshold approach

The significance of K^2^ was tested individually via a surrogate approach [43] according to the procedure reported in the Section “*Surrogate data approach for individual assessment of the K*^*2*^ *significance*” in the file Supplementary Material. The test was carried out over MAP and MCBv sequences of 256 consecutive values. Given that experimental recordings were longer than 256 consecutive values in any experimental condition (i.e., REST and STAND) and time point (i.e., PRE and POST), analyses were iterated overtime with an overlap of 255 values, namely with one beat shift at each iteration. The final marker was the median of the distribution of K^2^, TFP and TFG in each frequency band computed over the entire experimental session [44]. Two strategies were followed: (1) the median of the distribution was computed over all the sequences regardless of the value of K^2^; (2) the median of the distribution was computed after excluding those values whose K^2^ was not significant according to the surrogate test.

**Fig. 1:**
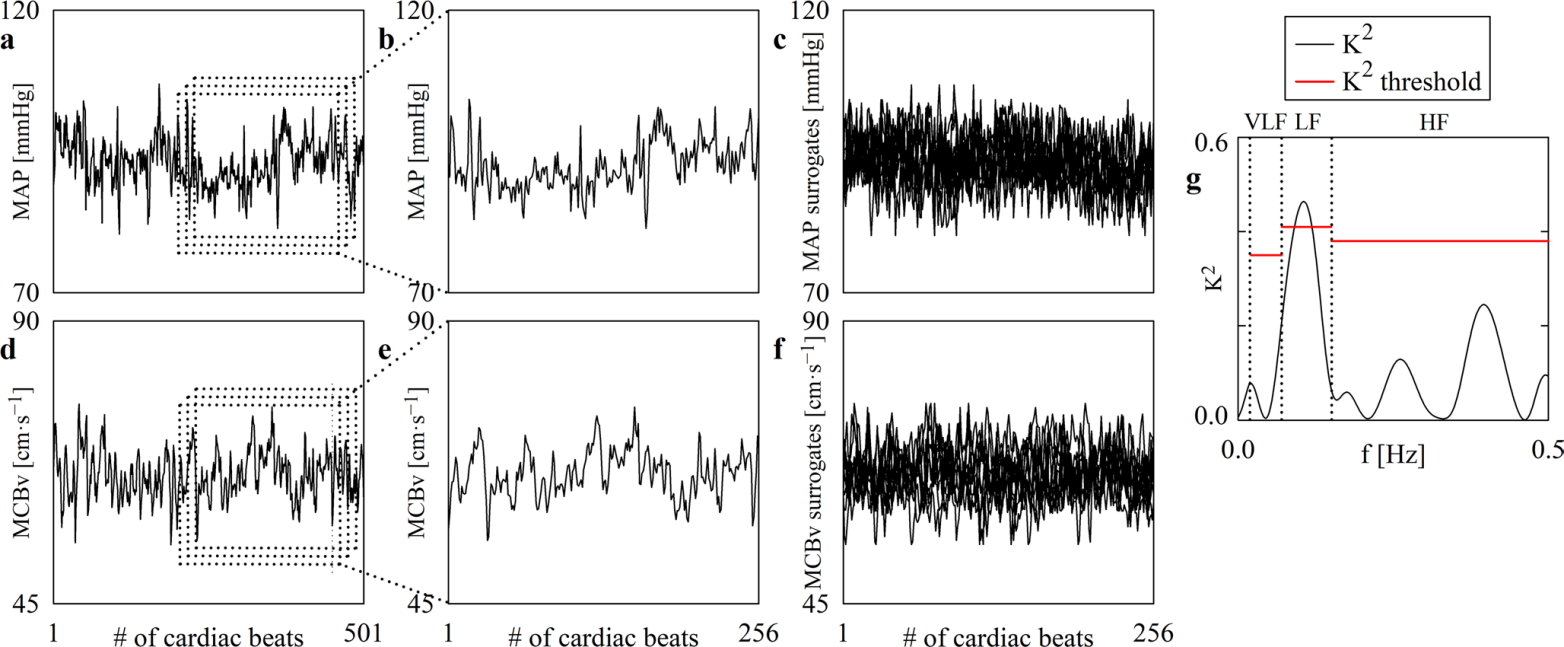
The line plots in (**a**) and (**d**) show, respectively, the time courses of MAP and MCBv variability series recorded from a representative subject at REST in PRE. The dotted rectangles indicated three representative frames of 256 consecutive values selected for the analysis according to the running strategy. The first frames of MAP and MCBv series are shown in (**b**) and (**e**) respectively. For each frame we generated 100 surrogate series. The first 10 surrogate series of MAP and MCBv relevant to the first frame are shown in (**c**) and (**f**). The solid black line in (**g**) represents the K^2^ computed over the original frames shown in (**b**) and (**e**), while the horizontal solid red lines represent the threshold of significance computed over the set of uncoupled surrogate pairs drawn in (**c**) and (**f**). The reported threshold of significance is relevant to the frequency at the maximum of K^2^ in the considered frequency band computed over the original series. The vertical dotted black lines in (g) indicate the limits of the VLF, LF and HF bands

Figure 1 exemplifies the running strategy adopted to build the threshold of K^2^. The original beat-to-beat series of MAP and MCBv, recorded at REST in PRE in a representative subject, are drawn in Figs. 1a, d respectively. The dotted black rectangles marked three frames of 256 consecutive values with overlap. The sequences of MAP and MCBv relevant to the first frame are expanded in Figs. 1b, e respectively. The first 10 surrogate series of MAP and MCBv generated from the original series reported in Figs. 1b, e are shown in Figs. 1c, f respectively. Figure 1g reports the course of K^2^ computed over the original series of MAP and MCBv shown in Figs. 1b, e as a solid black line. The threshold of K^2^ built over the set of surrogates relevant to the frequency at the maximum of K^2^ calculated over the original series in each frequency band are reported as horizontal solid red lines in Fig. 1g. The threshold varies with the frequency band (i.e., VLF, LF and HF bands). If the maximum value of K^2^ computed over the original series was above the threshold of significance, the null hypothesis of MCBv-MAP uncoupling was rejected for the considered frame of MAP and MCBv series. In Fig. 1g the null hypothesis of MCBv-MAP uncoupling was rejected in the LF band, and it was accepted in the VLF and HF ones. This check was iterated over the next dotted window reported in Figs. 1a, d, thus resulting in a running approach.

**Fig. 2:**
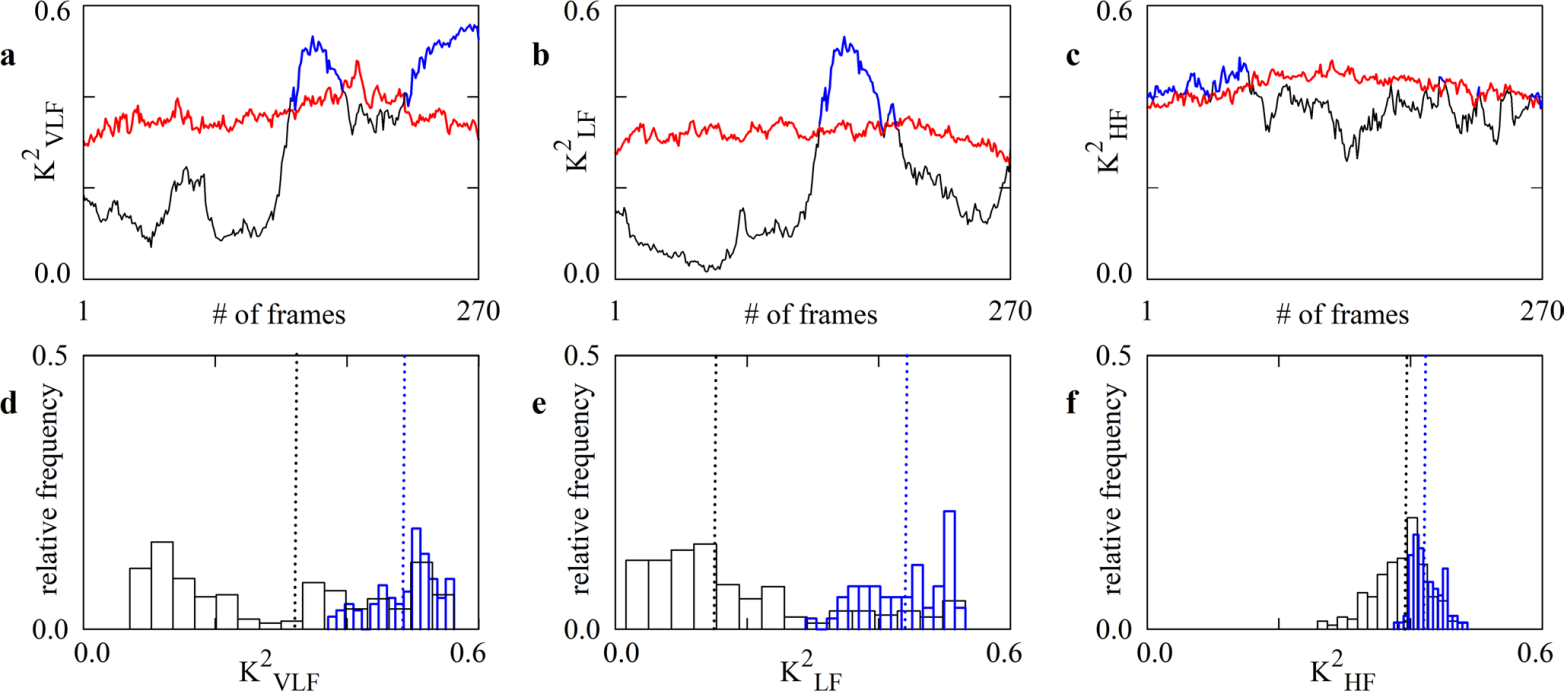
The line plots show the time course of K^2^ computed over original MAP and MCBv variability series (solid black line), the time course of the K^2^ threshold derived from the set of uncoupled surrogates (solid red line) and the values of K^2^ computed over original MAP and MCBv variability series above the threshold of significance (solid blue line) in (**a**), (**b**) and (**c**). Blue line covers partially the black line. Points of the curves were calculated in a representative subject at REST in PRE over frames of 256 consecutive values of MAP and MCBv and analysis was iterated overtime with an overlap of 255 values. The curves are relevant to K^2^ markers computed in VLF (a), LF (b), and HF (c) bands. In (**d**), (**e**) and (**f**) the distributions of K^2^ values computed over the original series regardless of the value of K^2^ are shown as black curves, while distributions computed over those values above the threshold of significance are shown as blue curves. Distributions are computed using 15 equal-size bins. The vertical dotted black and blue lines mark the median of the black and blue distributions of K^2^ values respectively

Figure 2 shows the result of a running analysis performed over a representative subject at REST in PRE. The length of the original MAP and MCBv series was 526 cardiac beats, thus leading to 270 analyses over frames of 256 cardiac beats with a superposition of 255 values. Figure 2 shows exclusively the results of the running analysis carried out over K^2^ but the threshold of K^2^ is utilized to exclude TFP and TFG data as well. K^2^ computed over the original pairs, the threshold of significance of K^2^ assessed from surrogate data, and the values of K^2^ computed over the original pairs above the threshold of significance were denoted as solid black, red and blue lines respectively. Blue line covers partially the black line. The distribution of the K^2^ values computed over the original pairs relevant to the VLF, LF and HF bands were shown in Figs. 2d, e,f respectively. The black distribution is relevant to all K^2^ values regardless of the level of K^2^, while the blue distribution is pertinent exclusively to those indexes above the K^2^ threshold. Medians of the black and blue distributions were indicated as vertical dotted black and blue lines respectively. Regardless of the frequency bands, K^2^ was generally weak (< 0.55) and for long periods of time it was below the threshold of significance (i.e., below the red line). On average K^2^ tended to be higher in HF band (Fig. 2f) than in VLF and LF bands (Figs. 2d, e). The medians computed over all the K^2^ values were 0.32 (Fig. 2d), 0.14 (Fig. 2e), and 0.39 (Fig. 2d), while those calculated solely over the K^2^ values above the threshold of significance were 0.49 (Fig. 2d), 0.44 (Fig. 2e), and 0.42 (Fig. 2d). The difference between medians of K^2^ values computed over all the frames and after the exclusion of the frames whose K^2^ values were below the threshold of significance could be large (Figs. 2d, e) or small (Fig. 2f).

### Statistical analysis

Two-way analysis of variance (Holm–Sidak test to account for the issue of multiple comparisons) was applied to time domain parameters, to the percentage of sequences whose K^2^ was above the significance threshold, and to the medians of TF-based markers to assess the impact of the orthostatic challenge within the same time point (i.e., PRE or POST) and the effect of the surgery within the same experimental condition (i.e., REST or STAND). Analyses over the median of TF-based markers were carried out separately over all the frames regardless of K^2^ and over those sequences whose K^2^ was above the significance threshold. If normality test (Shapiro-Wilk test) and equal variance test (Brown-Forsythe test) for the application of Holm-Sidak test were not passed, the Mann-Whitey rank sum test was applied. The level of significance of each Mann-Whitey rank sum test was divided by the number of comparisons (i.e., 4) to account for the multiple comparison issue. Statistical analysis was performed with a commercial statistical software (Sigmaplot v.14.0, Systat Software, San Jose, CA, USA). The level of statistical significance of all the tests was set to 0.05.

## Results

### Time- and frequency-domain univariate variability markers

Table 2 summarizes the time domain markers derived from HP, SAP, DAP, MAP and MCBv variability series at REST and during STAND in PRE and POST. µ_HP_ significantly decreased during STAND compared to REST in both PRE and POST and both at REST and during STAND µ_HP_ was lower in POST compared to PRE. In POST µ_DAP_ increased during STAND compared to REST and during STAND µ_DAP_ was higher in POST than in PRE. At REST σ^2^_HP_ was lower in POST with respect to PRE. Orthostatic challenge reduced µ_MCBv_ compared to REST, but this decrease was significant only in PRE.

**Table 2:** Time domain indexes of HP, SAP, DAP, MAP and MCBv variability series at REST and during STAND in PRE and POST

Index	Pre		Post	
	Rest	Stand	Rest	Stand
µ_HP_ [ms]	931 ± 112	836 ± 115 §	783 ± 119*	694 ± 113 §,*
σ^2^_HP_ [ms^2^]	1116 ± 1397	702 ± 633	371 ± 774*	246 ± 443
µ_SAP_ [mmHg]	142 ± 24	132 ± 20	134 ± 20	134 ± 21
σ^2^_SAP_ [mmHg^2^]	33 ± 23	39 ± 30	36 ± 25	45 ± 44
µ_DAP_ [mmHg]	70 ± 14	66 ± 15	70 ± 18	85 ± 22 §,*
σ^2^_DAP_ [mmHg^2^]	13 ± 9	15 ± 10	9 ± 7	26 ± 51
µ_MAP_ [mmHg]	96 ± 16	90 ± 15	90 ± 19	100 ± 21
σ^2^_MAP_ [mmHg^2^]	21 ± 14	24 ± 18	19 ± 14	32 ± 42
µ_MCBv_[cm·s^− 1^]	65 ± 28	53 ± 23 §	69 ± 34	58 ± 28
σ^2^_MCBv_ [cm^2^·s^− 2^]	39 ± 54	33 ± 36	42 ± 52	37 ± 37

*SAVR* surgical aortic valve replacement; *PRE* = before SAVR; *POST* within 7 days after SAVR; *REST* at rest in supine position; *STAND* during active standing; *HP* heart period; *AP* arterial pressure; *SAP* systolic AP; *DAP* diastolic AP; *MAP* mean AP; *MCBv* mean cerebral blood velocity; *µ* = mean; *σ*^2^ variance. *µ*_*HP*_, *µ*_*SAP*_, *µ*_*DAP*_, *µ*_*MAP*_, *µ*_*MCBv*_  µ of HP, SAP, DAP, MAP and MCBv series; *σ*^*2*^_*HP*_, *σ*^*2*^_*SAP*_, *σ*^*2*^_*DAP*_, *σ*^*2*^_*MAP*_, *σ*^*2*^_*MCBv*_ σ^2^ of HP, SAP, DAP, MAP and MCBv series. Data are reported as mean ± standard deviation. The symbol § indicates a significant difference between experimental conditions (i.e., REST and STAND) within the same time point (i.e., PRE or POST) with *p* < 0.05, while the symbol * marks a significant difference between time points within the same experimental condition with *p* < 0.05

Table 3 shows the frequency-domain univariate markers derived from MAP and MCBv variability series at REST and during STAND in PRE and POST. Neither orthostatic challenge nor time point induced significant changes.

**Table 3:** Frequency-domain indexes of MAP and MCBv variability series at REST and during STAND in PRE and POST

Index	Pre		Post	
	Rest	Stand	Rest	Stand
VLF_MAP_ [mmHg^2^]	2.0 ± 5.0	2.4 ± 4.8	1.9 ± 5.4	2.7 ± 7.6
LF_MAP_ [mmHg^2^]	2.2 ± 3.3	3.5 ± 9.7	1.0 ± 1.4	2.2 ± 4.7
HF_MAP_ [mmHg^2^]]	5.1 ± 4.7	4.3 ± 4.0	9.7 ± 10.6	8.8 ± 10.5
VLF_MCBv_ [cm^2^∙s^− 2^]	2.0 ± 3.9	3.2 ± 4.4	5.1 ± 8.7	4.2 ± 6.1
LF_MCBv_ [cm^2^∙s^− 2^]	10.0 ± 16.4	9.1 ± 14.8	5.5 ± 17.1	6.4 ± 15.4
HF_MCBv_ [cm^2^∙s^− 2^]	19.1 ± 37.4	15.6 ± 27.8	17.1 ± 26.2	18.2 ± 28.7

*SAVR* surgical aortic valve replacement; *PRE* before SAVR; *POST* within 7 days after SAVR; *REST* at rest in supine position; *STAND* during active standing; *MAP* mean arterial pressure; *MCBv* mean cerebral blood velocity; *VLF* very low frequency; *LF* low frequency; *HF* high frequency; *VLF*_*MAP*_, *LF*_*MAP*_, *HF*_*MAP*_ VLF, LF and HF powers of MAP series; *VLF*_*MCBv*_, *LF*_*MCBv*_, *HF*_*MCBv*_ VLF, LF and HF powers of MCBv series. Data are reported as mean ± standard deviation

### Frequency-domain bivariate CA markers

**Fig. 3:**
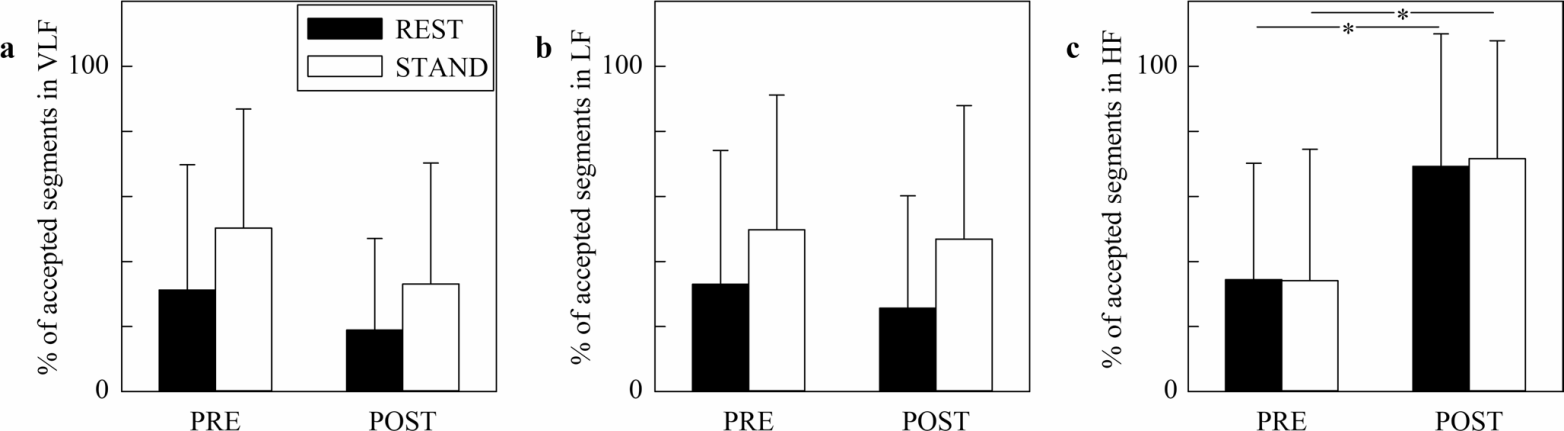
The grouped error bar graphs show the percentage of segments whose K^2^ is above the threshold of significance according to the surrogate approach, labelled as accepted sequences on the y-axis (**a**, **b**, **c**). Results are relevant to the VLF (a), LF (b), and HF (c) bands. Data are presented as mean + standard deviation. The symbol * marks a significant difference between time points (i.e., PRE and POST) within the same experimental condition (i.e., REST or STAND) with *p* < 0.05

Figure 3 shows the grouped error bar graphs relevant to the percentage of segments whose K^2^ value was above the K^2^ threshold (accepted sequences) as a function of the time point (i.e., in PRE and POST) at REST (black bars) and during STAND (white bars). Analysis was carried out in VLF (Fig. 3a), LF (Fig. 3b), and HF (Fig. 3c) bands. The percentage did not vary significantly across either time points or experimental conditions in VLF and LF bands (Figs. 3a, b), while in the HF band it increased after SAVR both at REST and during STAND (Fig. 3c).

**Fig. 4:**
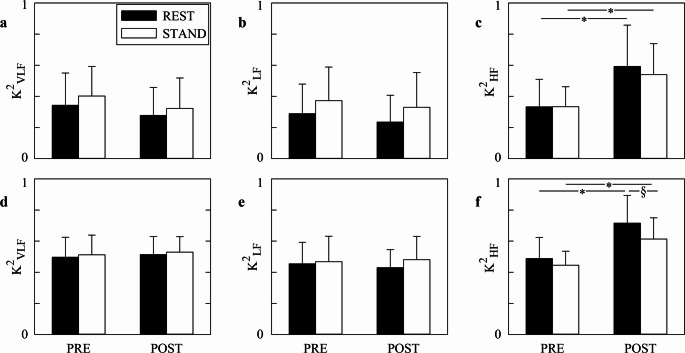
The grouped error bar graphs show the median of K^2^ values computed over all the sequences (**a**, **b**, **c**) and exclusively over those sequences featuring a significant value of K^2^ according to the surrogate test (**d**, **e**, **f**). Results are relevant to the VLF (a, d), LF (b, e), and HF (c, f) bands. Data are presented as mean + standard deviation. The symbol § indicates a significant difference between experimental conditions (i.e., REST and STAND) within the same time point (i.e., PRE or POST) with *p* < 0.05, while the symbol * marks a significant difference between time points within the same experimental condition with *p* < 0.05

Figure 4 shows the grouped error bar graphs relevant to median of K^2^ values computed over all the segments (Figs. 4a, b,c) and exclusively over the segments whose K^2^ was above the significance threshold according to the surrogate test (Figs. 4d, e,f) as a function of the time point at REST (black bars) and during STAND (white bars). Analysis was carried out in VLF (Figs. 4a, d), LF (Figs. 4b, e), and HF (Figs. 4c, f) bands. Regardless of the strategy followed to compute the median of K^2^ values, markers were similar across time points or experimental conditions in VLF and LF bands (Figs. 4a, b,d, e) and they increased after SAVR both at REST and during STAND in the HF band (Figs. 4c, f). Only the median of K^2^ values calculated over the segments whose K^2^ was above the significance threshold was smaller during STAND than at REST and this result held in POST (Fig. 4f).

**Fig. 5:**
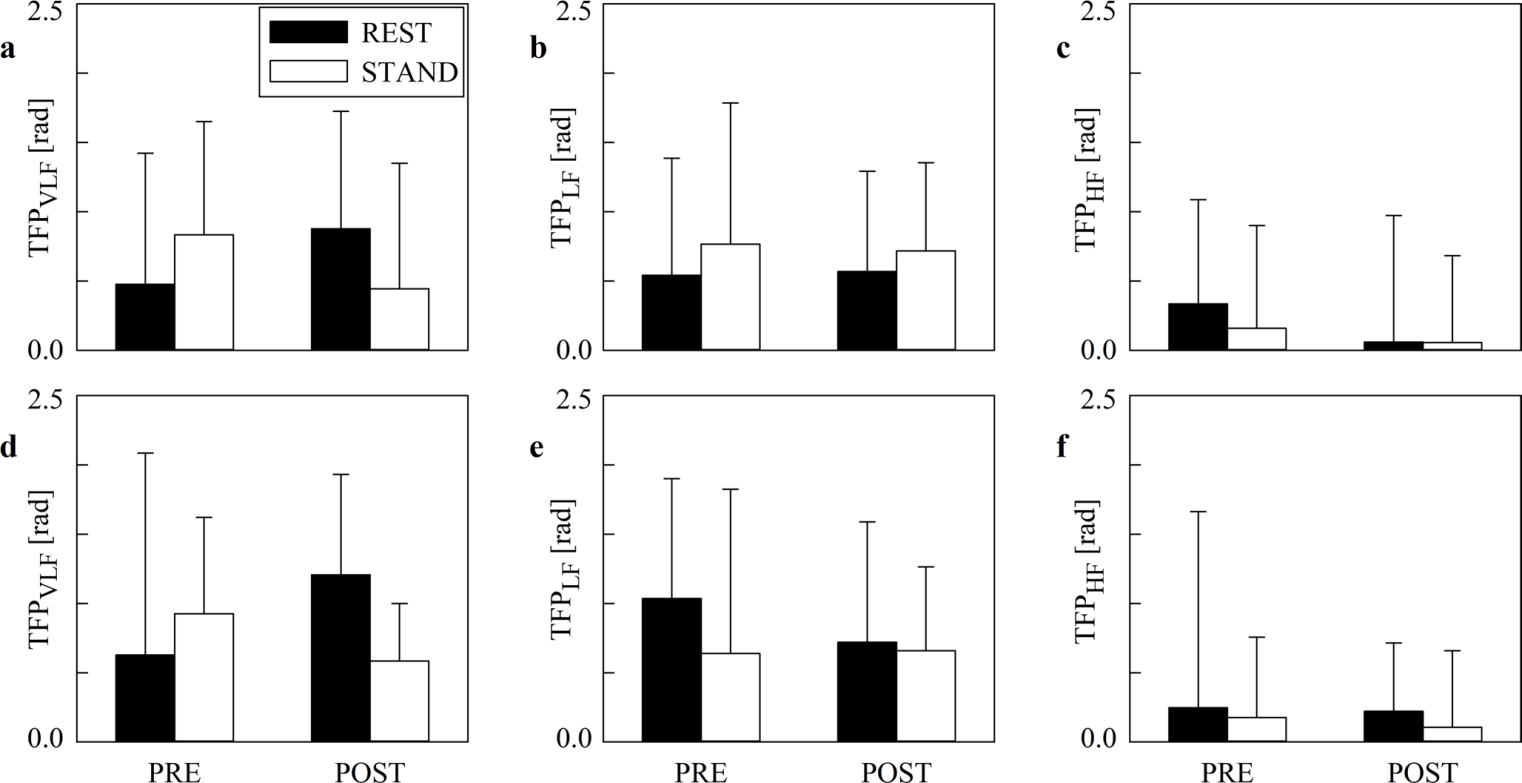
The grouped error bar graphs show the median of the TFP values computed over all the sequences (**a**, **b**, **c**) and exclusively over those sequences featuring a significant value of K^2^ according to the surrogate test (**d**, **e**, **f**). Results are relevant to the VLF (a, d), LF (b, e), and HF (c, f) bands. Data are presented as mean + standard deviation.

Figure 5 has the same structure as Fig. 4 but it shows the median of TFP values computed over all the segments regardless of the value of K^2^ (Figs. 5a, b,c) and exclusively over those sequences whose K^2^ was above the significance threshold according to surrogate test (Figs. 5d, e,f). The medians of the TFP did not change significantly across either time points or experimental conditions and this finding held regardless of the frequency bands.

**Fig. 6:**
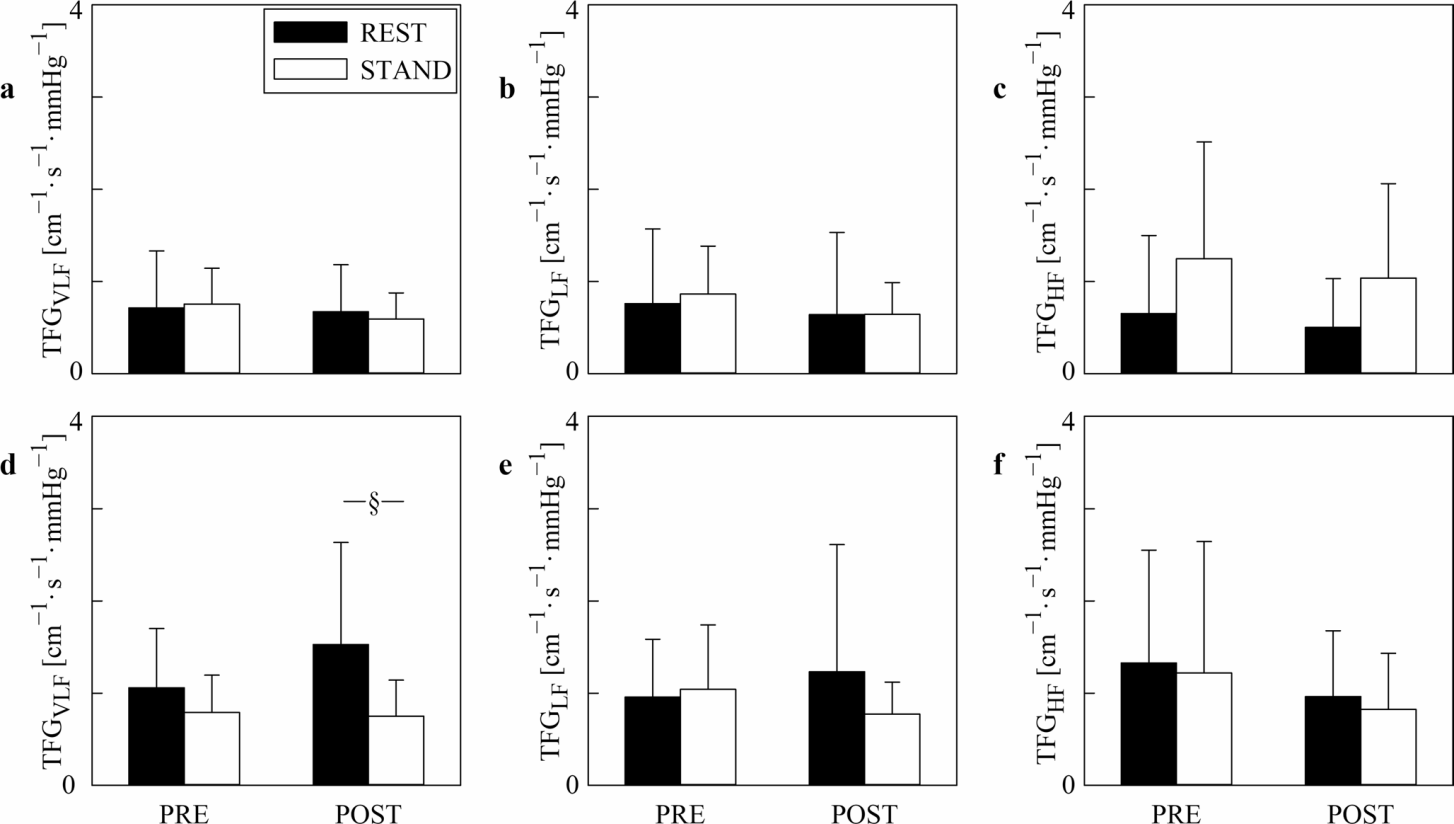
The grouped error bar graphs show the median of the TFG values computed over all the sequences (**a**, **b**, **c**) and exclusively over those sequences featuring a significant valuesof K^2^ according to the surrogate test (**d**, **e**, **f**). Results are relevant to the VLF (a, d), LF (b, e), and HF (c, f) bands. Data are presented as mean + standard deviation. The symbol § indicates a significant difference between experimental conditions (i.e., REST and STAND) within the same time point (i.e., PRE or POST) with *p* < 0.05

Figure 6 has the same structure as Figs. 4 and 5 but it shows the median of TFG values computed over all the segments (Figs. 6a, b,c) and exclusively over those sequences whose K^2^ was above the significance threshold according to surrogate test (Figs. 6d, e,f). The median of the TFG did not vary significantly across either time points or experimental conditions and this finding held regardless of the frequency bands, with the notable exception in the VLF band in POST where the median of the TFG values computed exclusively over those sequences whose K^2^ was above the significance threshold was lower during STAND compared to REST (Fig. 6d)

## Discussion

The main findings of the study can be summarized as follows: (i) we combined a surrogate approach that checked the significance of K^2^ on an individual basis and separately in each frequency band with a moving window strategy; (ii) the impact of excluding MAP and MCBv sequences whose K^2^ was below the threshold of significance was marginal; (iii) the preservation of CA before and after SAVR was confirmed even when considering only those sequences with a significant MCBv-MAP association.

### Rejecting the null hypothesis of MCBv-MAP uncoupling individually via a moving window approach

A theoretical requirement for the reliable assessment of the TF from an input series to an output one is the significant association between them [24–31]. This prerequisite was traditionally checked by assuming an arbitrarily high threshold of 0.5, indicating that at least 50% of the variance of the output can be explained by changes in the input at a given frequency. This strategy was utilized for a long time when the purpose was to estimate baroreflex sensitivity from spontaneous fluctuations of HP and SAP [24]. This approach was adequate in healthy subjects because the level of association between HP and SAP is commonly higher than 0.5, even though it decreases with age in the LF band [45]. Unfortunately, in populations under pharmacological challenges or exhibiting baroreflex impairment, the lower values of K^2^ made this requirement more difficult to fulfil [46, 47].

Theoretical approaches applied in association with a non-parametric technique for the assessment of K^2^ provide the threshold for K^2^ significance [48, 49]. These approaches derived a value that did not depend on the frequency under the hypothesis of independence of frames [48, 49]. These approaches were exploited in the context of assessing the significance of the MCBv-MAP association [3, 20]. These techniques allow an easier rejection of the null hypothesis of uncoupling because the threshold might be below 0.5 [20, 25, 46]. However, the use of a threshold invariable with frequency might be insufficient in the presence of dominant oscillations. Indeed, dominant oscillations, typically present in cardiovascular and cerebrovascular variability series [20, 25, 46], might prevent the fulfilment of hypothesis of independence between adjacent frames. Moreover, even in the case that the hypothesis of independence between adjacent frames was fulfilled due to the presence of a sufficient amount of random relative phase jitters between oscillations in MAP and MCBv at the same frequency, the detection of this situation might require large frame lengths to be safely confirmed, especially whether the dominant oscillations are slow. Given that short data sequences are commonly utilized in cardiovascular and cerebrovascular variability analyses, it is not surprising that the threshold for K^2^ significance obtained via surrogate technique is variable with frequency and raises in correspondence of the rate of the dominant oscillations for ensuring a safe rejection of the null hypothesis [26, 27]. The frequency-dependent behavior of the threshold for K^2^ significance in the presence of dominant oscillations was suggested using a parametric approach in association with the surrogate technique [26, 27]. We advocate studies comparing parametric and non-parametric approaches in the presence of dominant oscillations and in association with surrogate data to check whether the behavior of the threshold for K^2^ significance depends on the approach utilized to estimate the K^2^ function and whether the proposed approach could be applied even in association with non-parametric estimates of K^2^ function.

A threshold computed on an individual basis might be more appropriate because it could account for power distribution varying with experimental condition and frequency band [26, 27]. An individual threshold can be derived from a set of surrogates built specifically to destroy cross-correlation between the series, while preserving as much as possible the distribution of the values and power spectral density of the original series [50]. In the context of CA analysis, various surrogate-based strategies have been followed to check the significance of K^2^: (i) unmatched MAP and MCBv series were generated by low-pass filtering two independent gaussian white noise realizations [29]; (ii) original MAP series taken from one subject were associated to an original MCBv series taken from a different subject randomly chosen within the same group within the same experimental condition [28, 30]; (iii) uncoupled MAP and MCBv series were generated by time shifting the original version of the series using a random, sufficiently long, latency [40]; (iv) unmatched MAP and MCBv variability series were generated by randomizing Fourier phases of the original MAP and MCBv variability series with two independent uniformly-distributed realizations [31]. In the present contribution we followed the latter strategy [31]. The approach checks the significance of K^2^ individually and separately in VLF, LF and HF bands. The method was applied iteratively over the entire MAP and MCBv recordings, thus avoiding the selection of a unique frame within an experimental session. This procedure improves reproducibility of the analysis and prevents possible biases linked to the subjective selection of the analysis window. The medians of the TF-based markers computed over all the frames irrespectively of the value of K^2^ and solely over those sequences whose K^2^ value was above the significance threshold were calculated as final parameters undergoing statistical analysis.

Remarkably, the percentage of sequences whose K^2^ value was above the level set by uncoupled surrogates was low in both PRE and POST. This result was particularly evident in VLF and LF bands, thus stressing the low level of association between MAP and MCBv variability series in SAVR patients. This finding is expected in healthy subjects at REST [51] and it has been assumed to be an indication of a working CA given that one of the main aims of CA is to limit variability of MCBv against variations of MAP [3, 20]. Therefore, we conclude that in SAVR patients CA is preserved in both PRE and POST, thus confirming previous observations [17, 21, 22, 37, 52]. The invariance of the percentage of segments whose K^2^ values were above the threshold of significance during STAND compared to REST supports this conclusion. However, the low level of association between MAP and MCBv series might be linked to the presence of nonlinearities and/or noise contaminating variability series. Only in the HF band the percentage of sequences whose K^2^ values were above the significance threshold increased in POST compared to PRE and this result held both at REST and during STAND. This result is the consequence of the increase of K^2^ in POST compared to PRE in the HF band. This finding might be the result of the post-surgery improvement of the performance of the left ventricle [17, 37] that can convert more efficiently changes of intrathoracic pressure and venous return linked to respiration into modifications of the stroke volume and cardiac output [53, 54], thus driving MCBv variations more efficiently. Higher values of K^2^ between MAP and MCBv variability series were commonly detected in healthy subjects at REST in the HF band compared to VLF and LF bands [51]. The different results in the HF band compared to VLF and LF bands confirmed the necessity of distinguishing different frequency bands in this specific analysis [29] and the need of filtering to focus on a specific range of time scales when using time domain indexes based on cross-correlation technique [55–57].

### The impact of K^2^ threshold on the TF-based characterization of CA

The clinical exploitation of CA parameters is limited by their high variability and poor reproducibility [12, 15, 16]. Both physiological and methodological confounding factors are responsible for the low intra-subject and inter-subject agreement. Among the physiological confounding factors there are changes of autonomic activities [18], mechanical influences of respiration [58], and modifications of partial pressure of arterial carbon dioxide [59]. Among the methodological confounding factors reducing the statistical power of analysis there are the different reliability of TF-based markers according to different variability of MAP [16] and the variable degree of K^2^ between MAP and MCBv [3, 20]. In this study we tested the hypothesis that the exclusion of MAP and MCBv sequences whose K^2^ values were below a threshold individually set according to the proposed surrogate approach was more powerful in detecting differences across experimental conditions and time points in SAVR population compared to a strategy considering all the sequences regardless of the values of K^2^. Contrary to our expectation, exclusion of segments that did not feature a sufficient level of MCBv-MAP association did not improve the discriminative power of the analysis. This finding held regardless of frequency band and type of TF marker. The sole difference between the analyses carried out over all the sequences and solely over those frames with a significant degree of MCBv-MAP association was the more important decrease of K^2^_HF_ and TFG_VLF_ during STAND compared to REST in POST when the markers were computed by considering exclusively MAP and MCBv segments with a significant level of K^2^. One possible explanation of the marginal impact of accounting for the significance of K^2^ is that the cerebrovascular regulation is so complex, involving endothelial, metabolic, chemical, neurogenic, and myogenic factors, that testing this fundamental methodological prerequisite could be irrelevant in practice [60]. Indeed, accounting for the significance of K^2^ would have practical relevance only if the cross-influences between MAP and MCBv did not involve different frequencies, cross-effects between different components contributing to CA occurred at the same time scale and linear interactions were dominant. Conversely, since these expectations are unlikely due to nonlinear interactions and cross-scales interactions [41, 51], the application of a strategy based on the K^2^ threshold might have a limited efficacy. In addition, the role played by vascular properties of the pressure-to-flow link might be an additional confounding factor for the CA [61]. Our finding supports the notion that canceling confounding factors might improve the analysis more than accounting for the theoretical prerequisite of the significance of K^2^ [40, 58, 62]. Our data does not definitely prove the limited practical value of limiting the analysis exclusively to those frames featuring a significant value of K^2^, but this study suggests that the application of this procedure might not be helpful when changes of TF-based indexes between experimental conditions and/or time points are limited. Further studies are necessary to check whether there are experimental protocols where the application of this procedure reduces the dispersion of indexes and increases the statistical power of the analysis. We stress that conclusions of our study should be considered valid in the case of parametric estimates of K^2^ function [17, 22, 31, 37], while any extension to non-parametric approach should be validated [3, 20].

### CA is preserved in PRE and POST regardless of the outcome of the surrogate test

Our analysis carried out over MAP and MCBv frames regardless of the value of K^2^ confirms that CA is preserved in SAVR. Indeed, in PRE the orthostatic challenge could not modify TF-based indexes [17, 21, 22, 37, 52] and surgery did not importantly affect CA because markers remained unvaried in POST compared to PRE [17, 21, 22, 37, 52]. This conclusion was supported by the univariate frequency domain markers as well. Even the low percentages of frames whose K^2^ was above the significance level support the ability of cerebrovascular mechanisms to limit the impact of MAP variations. These findings are particularly evident in VLF and LF bands, namely over those temporal scales that are more pertinent to CA [3, 20, 51]. However, it might be argued that the limited impact of surgery and orthostatic challenge on TF-based markers in SAVR cohort is the trivial effect of noise reducing the value of K^2^. Conversely, this conclusion still holds even when the analysis of TF-based indexes was carried out solely over the sequences featuring a significant value of K^2^, thus limiting the role of additional factors on the conclusion about the CA preservation.

### Limitations of the study and future developments

The present study has some limitations. First, the cohort is relatively young and less compromised compared to the usual SAVR population. Older and more compromised people are less inclined to participate in the study due to the demanding protocol including orthostatic challenge. Second, some recordings are missing due to the difficulties in keeping the orthostatic position, especially in POST, as well as to the difficulty in insonating MCA during STAND. These difficulties might have contributed to increasing the dispersion of the results. Third, conclusions about the preservation of CA in PRE are based on the limited strength of the MCBv-MAP association and on the absence of its modifications during STAND. However, STAND might be insufficient to elicit modifications of the pressure-to-flow coupling strength. We advocate enlarging the size of the population to improve statistical power of the analysis and the inclusion of a suitable control group. Fourth, the analysis neglects the possible effect of nonstationaroties within the frame and the nonlinear interactions between MAP and MCBv that might have produced a bias toward lower values of K^2^. Future studies should apply tests for stationarity [63] and linearity [17] to exclude that conclusions might depend on the lack of these prerequisites for a reliable assessment of K^2^. Fifth, conclusions are valid for the considered frame length. Indeed, the threshold of significance depends on frame length because it plays an important role in setting the dispersion of K^2^ values at any frequency. However, the frame length is mainly decided according to the time scales of the cerebrovascular control mechanisms under scrutiny [3, 20]. Sixth, the time horizon of conclusions is limited because no follow-up was performed.

## Conclusions

We proposed a moving window approach based on iso-distribution iso-spectral uncoupled surrogate pairs to reject frames with a degree of association between MCBv and MAP variability series below a threshold of significance. The approach avoids a priori selection of the frame of analysis, works on an individual basis and allows independent tests in each frequency band. Accounting exclusively for frames whose K^2^ is above the significance level did not produce any additional advantage compared to considering all the frames regardless of K^2^ in the frequency bands typical of CA (i.e., VLF and LF), thus stressing the limited practical value of using K^2^ to select the most reliable frames for the characterization of CA in a context of limited inter-session variations TF-based indexes. The low and invariable percentages of frames with significance level of K^2^ as well as the trends of TF-based parameters across experimental conditions and time points of the analysis in the frequency bands typical of CA support the preservation of CA before and after SAVR. We conclude that testing the significance of K^2^ might provide marginal improvement when the aim is the characterization of a peculiar component to cerebrovascular regulation such as CA. Conclusion might be important because it was derived in a population and experimental conditions where the degree of the MCBv-MAP association is weak. In addition, the moving windows approach ensures the improvement of reproducibility of the results, the prevention of biases associated to the arbitrary selection of the analysis frame and the possibility to follow changes of the markers as a function of time.

## Supplementary Information

Below is the link to the electronic supplementary material.

Supplementary Material 1

## Data Availability

The datasets used in the present study are available from the corresponding author on reasonable request.
