# Supplementary material for: Accounting for squared coherence marginally improves the discriminative power of frequency domain cerebral autoregulation markers in surgical aortic valve replacement patients
Source: Med Biol Eng Comput. 2026 Apr 2;64(5):2001–16. doi: 10.1007/s11517-026-03558-4 (PMC13133193; doi:10.1007/s11517-026-03558-4)
Supplement: Supplementary file 1 — Supplementary Material 1 [file 11517_2026_3558_MOESM1_ESM.docx]

**Supplementary material of “Accounting for squared coherence marginally improves the discriminative power of frequency domain cerebral autoregulation markers in surgical aortic valve replacement patients”**

by Francesca Gelpi^*^, Beatrice Cairo^*^, Vlasta Bari,
Beatrice De Maria, Pavandeep Singh, Martina Anguissola,
Carlo De Vincentiis, Marianna Volpe, Raffaella Molfetta, Marco Ranucci, Alberto Porta

* F.G. and B.C. contributed equally to this work

Computation of the beat-to-beat variability series

The R-wave peaks were detected using a thresholding approach applied to the first derivative of the electrocardiogram. The *n*th heart period (HP) was derived as the time distance between the *n*th and the (*n*+1)th R-wave peaks. The *n*th systolic arterial pressure (SAP) and diastolic arterial pressure (DAP) values were identified as the local maximum of arterial pressure (AP) within the *n*th HP, and as the local minimum of AP following the *n*th SAP. The *n*th mean AP (MAP) was computed as the ratio of the definite integral of AP between the time occurrence of the (*n*–1)th and *n*th DAP values divided by the interdiastolic interval. Analogously we applied the same procedure to cerebral blood velocity (CBv) signal to compute mean CBv (MCBv), where the diastolic values of the CBv were detected as the local minima of CBv closest to the occurrences of (*n*–1)th and *n*th DAP values [1]. The resulting series were manually checked and corrected in the case of missing cardiac beats or misdetections. The effect of ectopic beats or isolated arrhythmic events were mitigated via linear interpolation between the closest values unaffected by arrhythmic beat. Corrections did not exceed 5% of the total sequence length. Several frequency-domain univariate and bivariate indexes were computed from MAP and MCBv variability series for the characterization of cerebral autoregulation (CA).

Frequency-domain univariate CA markers

Frequency-domain univariate markers were calculated over MAP and MCBv variability series according to an autoregressive (AR) modeling approach. AR model describes the current value of the series as a linear combination of past values weighted by constant coefficients plus the current sample of a realization of an innovation process modelled as a white noise [2]. The Levinson-Durbin recursion was exploited to identify the coefficients of the linear regression and the variance of the white noise. The power spectral density can be computed from the transfer function (TF) of the AR model and the variance of the white noise [2]. The AR model order was optimized via the Akaike's information criterion [3] in the range from 8 to 14 [4]. Residue theorem was utilized to decompose the AR power spectral density into spectral components associated to a real pole or a pair of complex conjugate poles of the TF of the AR model [5]. Spectral indexes were computed by summing the power of all spectral components associated to poles whose central frequency fall into the very low frequency (VLF) band (from 0.02 to 0.07 Hz), low frequency (LF) band (from 0.07 to 0.15 Hz) and high frequency (HF) band (from 0.15 to 0.5 Hz) [6,7].

Frequency-domain bivariate CA markers

Frequency-domain bivariate indexes were based on the computation of the TF from MAP to MCBv variability series according to a bivariate AR (BAR) modeling approach. BAR model describes the current value of the output series as a linear combination of past values of the same series and of the input series weighted by constant coefficients plus the current sample of a realization of an innovation process modelled as a white noise [8]. TF was computed as the ratio of the power cross-spectral density from MAP to MCBv to the power spectral density of MAP [9,10]. TF was computed over the range of frequencies typical of spontaneous variability (*i.e.*, 0 ≤ *f* ≤ 0.5 Hz). The TF gain (TFG) was computed as the modulus of the TF. The TF phase (TFP) was assessed as the phase of the power cross-spectral density. The squared coherence (K^2^) function was calculated as the ratio of the square modulus of the power cross-spectral density from MAP to MCBv to the product of the power spectral densities of the MCBv and MAP variability series [9,10]. The BAR model order was optimized via the Akaike information criterion for multivariate processes in the range from 5 to 12 [4] and the coefficients of the model were identified via the least squares method solved via Cholesky factorization technique [8]. The TFG, TFP, and K^2^ were sampled at the peak of K^2^ detected in the VLF, LF, and HF bands. The indexes were labelled TFG_VLF_, TFP_VLF_, K^2^_VLF_, TFG_LF_, TFP_LF_, K^2^_LF_, TFG_HF_, TFP_HF_, and K^2^_HF_. TFG indexes represent the magnitude of the MCBv changes per unit modification of MAP. TFG indexes were expressed in cm·s^−1^·mmHg^−1^. TFP ranged from −π and +π and was expressed in radians (rad). Positive values indicated that MCBv changes led MAP variations. K^2^ indexes measured the strength of the linear coupling between MAP and MCBv being in between 0 and 1, where 0 represents null association and 1 indicates perfect association, respectively.

Surrogate approach for individual assessment of the K^2^ significance

The significance of K^2^ was tested via a surrogate approach [11]. For each pair of original MAP and MCBv sequences we built 100 pairs of surrogate MAP and MCBv sequences preserving as much as possible the dynamic properties of the original series but being fully uncoupled. The length *N* of the sequence was 256. We exploited iteratively-refined amplitude-adjusted Fourier transform-based (IAAFT) approach [12] to build pairs of MAP and MCBv series with the same distribution as the original series, while power spectral densities were the best approximation according to the number of iterates (*i.e.*, 100). Since in the IAAFT method the phases of the Fourier transform of the original MAP and MCBv series were substituted with realizations of a uniformly distributed random process ranging from 0 to 2π, the use of two independent sequences generated according to two different random seeds, made the two surrogate series of MAP and MCBv fully uncoupled [13]. The length *N* of the series allowed us to speed up the construction of surrogate data via fast Fourier transform. K^2^ markers were computed over the set of surrogates and the 95th percentile was extracted at any frequency. We built the 95th percentile of the K^2^ distribution derived from surrogates. If the maximum of the K^2^ computed over the original series within the considered band was above the maximum of the K^2^ threshold computed in the same band, the null hypothesis of uncoupling was rejected and the alternative hypothesis, namely MAP and MCBv variability series were significantly associated, was accepted [14].

Type-I probability values relevant to Table 2 and Table 3

Below we report in Table 4 and Table 5 the values of type-I error probability relevant to Table 2 and Table 3.

**Table 4.** Type-I error probability of comparisons of time domain HP, SAP, DAP, MAP and MCBv indexes reported in Table 2.

| **Index** | **STAND vs REST in PRE** | **STAND vs REST in POST** | **POST vs PRE at REST** | **POST vs PRE during STAND** |
| --- | --- | --- | --- | --- |
| μ_HP_ [ms] | <0.001 | <0.001 | <0.001 | <0.001 |
| σ^2^_HP_ [ms^2^] | 0.167 | 0.208 | 0.018 | 0.063 |
| μ_SAP_ [mmHg] | 0.206 | 0.267 | 0.338 | 0.474 |
| σ^2^_SAP_ [mmHg^2^] | 0.681 | 0.239 | 0.752 | 0.313 |
| μ_DAP_ [mmHg] | 0.638 | 0.01 | 0.98 | 0.037 |
| σ^2^_DAP_ [mmHg^2^] | 0.934 | 0.076 | 0.562 | 0.152 |
| μ_MAP_ [mmHg] | 0.317 | 0.123 | 0.212 | 0.446 |
| σ^2^_MAP_ [mmHg^2^] | 0.976 | 0.137 | 0.71 | 0.141 |
| μ_MCBv_[cm·s^−1^] | 0.002 | 0.066 | 0.463 | 0.285 |
| σ^2^_MCBv_ [cm^2^·s^−2^] | 0.527 | 0.241 | 0.489 | 0.480 |

SAVR = surgical aortic valve replacement; PRE = before SAVR; POST = within 7 days after SAVR; REST = at rest in supine position; STAND = during active standing; HP = heart period; AP = arterial pressure; SAP = systolic AP; DAP = diastolic AP; MAP = mean AP; MCBv = mean cerebral blood velocity; μ = mean; σ^2^ = variance. μ_HP_, μ_SAP_, μ_DAP_, μ_MAP_, μ_MCBv_ = μ of HP, SAP, DAP, MAP and MCBv series; σ^2^_HP_, σ^2^_SAP_, σ^2^_DAP_, σ^2^_MAP_, σ^2^_MCBv_ = σ^2^ of HP, SAP, DAP, MAP and MCBv series

**Table 5.** Type-I error probability of comparisons of frequency domain HP, SAP, DAP, MAP and MCBv indexes reported in Table 3.

| **Index** | **STAND vs REST in PRE** | **STAND vs REST in POST** | **POST vs PRE at REST** | **POST vs PRE during STAND** |
| --- | --- | --- | --- | --- |
| VLF_MAP_ [mmHg^2^] | 0.421 | 0.163 | 0.86 | 0.294 |
| LF_MAP_ [mmHg^2^] | 0.514 | 0.986 | 0.462 | 0.289 |
| HF_MAP_ [mmHg^2^]] | 0.442 | 0.623 | 0.075 | 0.115 |
| VLF_MCBv_ [cm^2^∙s^−2^] | 0.408 | 0.491 | 0.093 | 0.844 |
| LF_MCBv_ [cm^2^∙s^−2^] | 0.229 | 0.09 | 0.325 | 0.401 |
| HF_MCBv_ [cm^2^∙s^−2^] | 0.678 | 0.579 | 0.48 | 0.787 |

SAVR = surgical aortic valve replacement; PRE = before SAVR; POST = within 7 days after SAVR; REST = at rest in supine position; STAND = during active standing; MAP = mean arterial pressure; MCBv = mean cerebral blood velocity; VLF = very low frequency; LF = low frequency; HF = high frequency; VLF_MAP_, LF_MAP_, HF_MAP_ = VLF, LF and HF powers of MAP series; VLF_MCBv_, LF_MCBv_, HF_MCBv_ = VLF, LF and HF powers of MCBv series.

References

[1] Bari V, Marchi A, De Maria B, Rossato G, Nollo G, Faes L et al (2016) Nonlinear effects of respiration on the crosstalk between cardiovascular and cerebrovascular control systems. Phil Trans R Soc A Math, Phys Eng Sci 374(2067):20150179. doi: 10.1098/rsta.2015.0179.

[2] Kay SM, Marple SL (1981) Spectrum analysis: A modern perspective. Proc IEEE 69(11):1380–1418. doi: 10.1109/PROC.1981.12184.

[3] Akaike H (1974) A new look at the statistical novel identification. IEEE Trans Autom Control 19(6):716–723. doi: 10.1109/TAC.1974.1100705.

[4] Baselli G, Porta A, Rimoldi O, Pagani M, Cerutti S (1997) Spectral decomposition in multichannel recordings based on multi-variate parametric identification. IEEE Trans Biomed Eng 44(11):1092–1101. doi: 10.1109/10.641336.

[5] Porta A, Fantinato A, Bari V, Gelpi F, Cairo B, De Maria B et al (2020) Evaluation of the impact of surgical aortic valve replacement on short-term cardiovascular and cerebrovascular controls through spontaneous variability analysis. PLoS ONE 15(12):e0243869. doi: 10.1371/journal.pone.0243869.

[6] Claassen JA, Meel-van den Abeelen AS, Simpson DM, Panerai RB (2016) Transfer function analysis of dynamic cerebral autoregulation: a white paper from the International Cerebral Autoregulation Research Network. J Cereb Blood Flow Metab 36(4):665–680. doi: 10.1177/0271678X15626425.

[7] Vaini E, Bari V, Fantinato A, Pistuddi V, Cairo B, De Maria B et al (2019) Causality analysis reveals the link between cerebrovascular control and acute kidney dysfunction after coronary artery bypass grafting. Physiol Meas 40(6):064006. doi: 10.1088/1361-6579/ab21b1.

[8] Porta A, Baselli G, Rimoldi O, Malliani A, Pagani M (2000) Assessing baroreflex gain from spontaneous variability in conscious dogs: role of causality and respiration. Am J Physiol Heart Circ Physiol 279(5):H2558–H2567. doi: 10.1152/ajpheart.2000.279.5.H2558.

[9] Zhang R, Zuckerman JH, Giller CA, Levine BD (1998) Transfer function analysis of dynamic cerebral autoregulation in humans. Am J Physiol 274(1 Pt 2):H233–H241. doi: 10.1152/ajpheart.1998.274.1.h233.

[10] Bendat JS, Piersol AG (2010) Random data: analysis and measurement procedures. Fourth Edition, John Wiley & Sons, Hoboken, New Jersey, USA.

[11] Prichard D, Theiler J (1994) Generating surrogate data from time series with several simultaneously variables. Phys Rev Lett 73(7):951–954. doi: 10.1103/PhysRevLett.73.951.

[12] Schreiber T, Schmitz A (1996) Improved surrogate data for nonlinearity tests. Phys Rev Lett 77:635–638. doi: 10.1103/PhysRevLett.77.635.

[13] Palus M (1997) Detecting phase synchronisation in noisy systems. Phys Lett A 235(4):341–351. doi: 10.1016/S0375-9601(97)00635-X.

[14] Porta A, Gelpi F, Bari V, Cairo B, De Maria B, Tonon D et al (2023) Concomitant evaluation of cardiovascular and cerebrovascular controls via Geweke spectral causality to assess the propensity to postural syncope. Med Biol Eng Comput 61(12):3141–3157. doi: 10.1007/s11517-023-02885-0.
